# Supplementary material for: Selective clearance of the inner nuclear membrane protein emerin by vesicular transport during ER stress
Source: eLife. 2019 Oct 10;8:e49796. doi: 10.7554/eLife.49796 (PMC6802967; doi:10.7554/eLife.49796)
Supplement: Transparent reporting form [file elife-49796-transrepform.pdf]

eLIFE
Westbrook Centre, Milton Road
Cambridge CB4 1YG
UK
P 01223 855340
W elifesciences.org
T @elife

eLife's transparent reporting form

We encourage authors to provide detailed information *within their submission* to facilitate the interpretation and replication of experiments. Authors can upload supporting documentation to indicate the use of appropriate reporting guidelines for health-related research (see EQUATOR Network), life science research (see the BioSharing Information Resource), or the ARRIVE guidelines for reporting work involving animal research. Where applicable, authors should refer to any relevant reporting standards documents in this form.

If you have any questions, please consult our Journal Policies and/or contact us: editorial@elifesciences.org.

Sample-size estimation

- You should state whether an appropriate sample size was computed when the study was being designed
- You should state the statistical method of sample size computation and any required assumptions
- If no explicit power analysis was used, you should describe how you decided what sample (replicate) size (number) to use

Please outline where this information can be found within the submission (e.g., sections or figure legends), or explain why this information doesn't apply to your submission:

For proteomic pulse-chase experiments, we followed the standard of the field, which considers each timepoint and independent measurement of labeled vs. unlabeled protein abundance. Importantly, detection of multiple peptides increases confidence and statistical power of the data when pooled together to determine protein-level half-lives. A detailed description of proteomic data analysis can be found in our Methods section; numbers of peptides detected for half-life determination can be found in our supplementary data.

For microscopy experiments, we followed the standard of the field and imaged a minimum of ~40 cells over several replicate experiments (see below).

No power analysis was performed to determine sample sizes needed.

Replicates

- You should report how often each experiment was performed
- You should include a definition of biological versus technical replication
- The data obtained should be provided and sufficient information should be provided to indicate the number of independent biological and/or technical replicates
- If you encountered any outliers, you should describe how these were handled
- Criteria for exclusion/inclusion of data should be clearly stated
- High-throughput sequence data should be uploaded before submission, with a private link for reviewers provided (these are available from both GEO and ArrayExpress)

Please outline where this information can be found within the submission (e.g., sections or figure legends), or explain why this information doesn't apply to your submission:

eLife Sciences Publications, Ltd is a limited liability non-profit non-stock corporation incorporated in the State of Delaware, USA, with company number 5030732, and is registered in the UK with company number FC030576 and branch number BR015634 at the address Westbrook Centre, Milton Road Cambridge CB4 1YG, UK | March 2019

Proteomic data are shown for 4 independent timepoints from 1 representative experiment. We have repeated this entire pulse-chase experiment and observed consistent results. Individual peptides that did not meet quality metrics were filtered out of our analyses; the rationale for these filtering steps is explained in our Methods section and in our analysis script that has been uploaded onto Dryad.

All microscopy experiments were performed on a minimum of 2 technical replicates and 2 biological replicates. We define technical replicates as duplicate wells of an imaging chamber that were treated identically; we define a biological replicate as a group of samples that were prepared and processed on a different day. Sample sizes vary from ~40 cells to >400 cells across conditions; the minimum N across a group of conditions that are compared to each other is reported in each figure legend. No outliers were excluded from these analyses.

Statistical reporting

- Statistical analysis methods should be described and justified
- Raw data should be presented in figures whenever informative to do so (typically when N per group is less than 10)
- For each experiment, you should identify the statistical tests used, exact values of N, definitions of center, methods of multiple test correction, and dispersion and precision measures (e.g., mean, median, SD, SEM, confidence intervals; and, for the major substantive results, a measure of effect size (e.g., Pearson's r, Cohen's d)
- Report exact p-values wherever possible alongside the summary statistics and 95% confidence intervals. These should be reported for all key questions and not only when the p-value is less than 0.05.

Please outline where this information can be found within the submission (e.g., sections or figure legends), or explain why this information doesn’t apply to your submission:

This information is included in each figure legend.

(For large datasets, or papers with a very large number of statistical tests, you may upload a single table file with tests, Ns, etc., with reference to sections in the manuscript.)

Group allocation

- Indicate how samples were allocated into experimental groups (in the case of clinical studies, please specify allocation to treatment method); if randomization was used, please also state if restricted randomization was applied
- Indicate if masking was used during group allocation, data collection and/or data analysis

Please outline where this information can be found within the submission (e.g., sections or figure legends), or explain why this information doesn’t apply to your submission:

N/A

Additional data files ("source data")

- We encourage you to upload relevant additional data files, such as numerical data that are represented as a graph in a figure, or as a summary table
- Where provided, these should be in the most useful format, and they can be uploaded as "Source data" files linked to a main figure or table
- Include model definition files including the full list of parameters used
- Include code used for data analysis (e.g., R, MatLab)
- Avoid stating that data files are "available upon request"

Please indicate the figures or tables for which source data files have been provided:

We have uploaded source data for Figure 1. This includes: raw unfiltered peptide data; our analysis pipeline (in a commented R script); filtered peptides (Supplementary Table 1); filtered proteins (Supplementary Table 2); results of half-life fitting (Supplementary Tables 3 and 4); and highlighted data for selected proteins of interest (Supplementary Table 5).
